# Data on the sensory evaluation of the dry red and white wines quality obtained by traditional technologies from European and hybrid grape varieties in the Krasnodar Territory, Russia

**DOI:** 10.1016/j.dib.2021.106992

**Published:** 2021-03-23

**Authors:** Alexan A. Khalafyan, Zaual A. Temerdashev, Vera A. Akin'shina, Yuri F. Yakuba

**Affiliations:** a Kuban State University, 149 Stavropol'skaya St., Krasnodar, 350040 Russia; b North Caucasian Federal Research Center of Horticulture, Viticulture, Wine-making, Russia

**Keywords:** Wine tasting, Expert evaluation of data, Positional analysis

## Abstract

The analysis of data on the sensory evaluation of the quality of wines obtained using traditional technologies in the Krasnodar Territory, Russia, was carried out using the statistical ranking criteria – the Spearman and Kendall correlation coefficients, as well as the positional analysis – Cronbach's alpha. Data on the sensory evaluation of 60 samples of natural dry red and white wines are presented, among which 20 are white wines, 40 are red wines produced in 2010–2015. Eleven specialists aged between 32 and 66 years (the average age was 50 years; 4 females and 7 males) participated in the sensory evaluation procedure. All participants are considered experts in the field of wine, work in the wine industry and have professional experience in the field of sensory analysis. The results of the consistency study of expert evaluations, the reliability of the general score scale, as well as the analysis of the loyalty of experts in the wine quality assessment are presented in the article. The reliability of the proposed loyalty scale is shown, i.e., the scale of the sum of scores given by each expert in the evaluation of the quality of wines. The database on the sensory evaluation of the quality of wines, obtained for all wine samples using positional analysis, makes it possible to assess the contribution of each of the 60 wine samples to their ranking by mean scores. The data may be of interest to scientists and oenologists for the wine quality assessment.

## Specifications Table

**Table:** 

Subject	Oenology
Specific subject area	Sensory analysis
Type of data	Figures, tables
How data were acquired	Data analysis
Data format	Results of the sensory evaluation of tested wine samples
Parameters for data collection	60 samples of natural dry red and white grape wines produced in 2010–2015 were analyzed. All the wine samples were produced according to traditional technologies from European (Cabernet, Merlot, Aligote, Riesling, Saperavi, etc.) and hybrid grape varieties (Bianca, Viorica, Moldova, Pervenets Magaracha, etc.).
Description of data collection	Samples of natural red and white wines were analyzed, among which the first 20 were white wines, the remaining 40 were red. Eleven specialists aged between 32 and 66 years (the average age was 50 years; 4 females and 7 males) participated in the sensory evaluation procedure. All participants are considered experts in the field of wine, work in the wine industry and have professional experience in the field of sensory analysis.
Data source location	The wines were produced in 2010–2015 in the Krasnodar Territory, Russia by industrial producers (alcohol content – 9–13% by volume, acidity – 4–7 g/dm^3^).
Data accessibility	Data available in the article
Related research article	A. A. Khalafyan, Z. A. Temerdashev, V. A. Akin'shina, Yu. F. Yakuba. Study of consistency of expert evaluations of wine sensory characteristics by positional analysis. *Heliyon*. 7(2) (2021) e06162. https://doi.org/10.1016/j.heliyon.2021.e06162

## Value of the Data

• The data provide insight into the problems and solutions of statistical analysis of the sensory evaluation and establishing the consistency of expert evaluations of wine quality.
• Comparing to the traditionally applied Spearman correlation coefficient and Kendall coefficient of concordance, the Cronbach alpha criterion of the positional analysis is calculated using the initial score scale taking into account its variability and allowing to evaluate the contribution of each expert to the consistency of expert evaluations and determine the reliability of the total score scale for each wine sample.
• The data can be compared with publications of other authors and/or used in comparative analysis and expert evaluation of the quality of wines.

## 1 Data Description

Data processing of the sensory evaluation of wine quality has been carried out by various statistical methods – analysis of variance (ANOVA) [1], [2], [3], principal component analysis (PCA) [4], discriminant analysis [5], mapping on the Cartesian plane [6], regression analysis [7,8], statistical text analysis using Alceste [9], etc. Expert methods for data processing, which describe the procedure for the sensory evaluation of wines [3,[10], [11], [12], [13], [14], [15], have a number of limitations. The results of the sensory evaluation of wines are influenced by the composition of experts, their qualification level and quantity as well as imbalance of wines. Individual characteristics inherent in each expert along with their physical and psycho-emotional state also contribute to the subjectivity of expert evaluations. In the present paper, the problems associated with analyzing the consistency of expert evaluations of wine quality, establishing the contribution of each expert to the total consistency and reliability of the total score scale for wine samples set by each expert have been considered. To process expert evaluations, Table 1 was created containing the scores set by 11 experts based on the results of organoleptic evaluation of 60 samples of white (samples 1–20) and red dry (samples 21–60) wines. The top row contains the number of experts, the first column is the sample number, the second and subsequent columns are expert scores of the wine quality, the last column is the sum of expert scores. The calculations were conducted using the STATISTICA software [16].

**Table 1:** Results of the sensory evaluation of tested wine samples.

Sample Number	Expert 1	Expert 2	Expert 3	Expert 4	Expert 5	Expert 6	Expert 7	Expert 8	Expert 9	Expert 10	Expert 11	Sum
1	77	80	80	80	**81**	82	77	85	85	83	81	891
2	83	83	79	79	**79**	63	82	79	83	78	78	866
3	89	79	81	83	**82**	76	83	79	86	77	85	900
4	90	85	82	78	**82**	78	85	76	85	84	78	903
5	90	87	79	85	**85**	82	84	83	85	85	85	930
6	90	84	80	84	**83**	76	77	86	83	81	84	908
7	85	87	82	85	**84**	78	83	86	84	86	87	927
8	90	86	80	83	**84**	84	83	87	82	82	79	920
9	95	92	85	84	**86**	80	88	88	84	84	80	946
10	88	86	82	79	**81**	64	85	80	83	79	86	893
11	83	79	79	78	**77**	58	77	72	86	74	81	844
12	82	86	82	82	**84**	84	84	82	87	85	84	922
13	88	85	87	80	**82**	72	84	73	84	83	86	904
14	88	87	80	79	**82**	72	80	90	85	80	83	906
15	94	88	80	86	**84**	80	88	81	83	79	82	925
16	82	90	82	80	**83**	82	82	78	86	82	83	910
17	84	79	78	79	**79**	72	78	78	82	77	83	869
18	87	83	79	79	**81**	74	88	77	83	80	81	892
19	84	82	79	78	**79**	71	77	72	83	86	82	873
20	95	89	82	86	86	84	89	83	85	79	86	944
21	87	83	80	82	84	80	79	81	81	79	82	898
**22**	**67**	**78**	**68**	**70**	**78**	**74**	**81**	**30**	**60**	**67**	**68**	**741**
23	88	81	79	79	82	84	83	75	78	71	82	882
24	85	88	80	79	85	84	81	80	82	78	86	908
25	81	80	79	82	81	84	84	86	78	82	81	898
26	92	83	80	85	84	86	84	82	80	80	84	920
27	85	86	79	78	86	88	77	76	82	74	82	893
28	87	83	79	86	85	78	79	82	85	78	81	903
29	82	82	80	83	85	81	83	88	78	76	81	899
30	87	84	81	81	83	82	85	80	82	84	87	916
31	79	90	80	79	82	80	83	80	80	80	86	899
32	83	89	79	82	84	88	82	92	78	82	84	923
33	81	89	80	78	84	90	77	81	82	84	81	907
34	81	86	80	84	83	82	83	85	75	88	83	910
35	89	91	82	84	85	82	82	82	86	77	84	924
36	87	89	81	81	84	86	84	92	82	82	86	934
37	87	90	83	82	81	84	85	79	79	85	84	919
38	85	80	80	81	81	78	81	88	77	79	79	889
39	94	82	81	80	84	86	84	81	81	83	80	916
40	87	91	82	80	85	82	85	92	82	89	83	938
41	82	89	82	82	85	84	89	84	78	87	81	923
42	79	78	**77**	78	82	80	86	60	78	79	70	847
43	84	87	**81**	78	86	82	84	73	78	82	80	895
44	87	82	**82**	85	85	82	78	80	83	75	85	904
45	86	92	**86**	87	86	93	84	89	83	79	79	944
46	88	89	**86**	80	83	94	83	89	84	87	86	949
47	82	79	**84**	80	84	92	86	85	86	88	79	925
48	85	85	**85**	83	83	91	86	88	80	83	81	930
49	86	93	**88**	88	84	92	88	95	78	88	90	970
50	87	84	**83**	84	86	81	89	80	78	80	83	915
51	84	86	**84**	80	85	84	85	86	83	82	80	919
52	75	87	**83**	84	83	82	83	78	85	78	90	908
53	80	80	**82**	82	84	88	78	83	88	80	80	905
54	85	79	**84**	84	83	86	82	86	85	86	87	927
55	86	89	**89**	89	94	84	90	93	88	90	83	975
56	75	80	**80**	79	80	82	81	80	79	78	81	875
57	88	85	**82**	86	82	84	77	80	81	81	79	905
58	84	87	**83**	87	83	80	83	80	79	80	82	908
59	84	82	**82**	87	83	87	85	80	80	76	80	906
60	70	78	**76**	70	78	76	89	72	79	76	70	834

The obtained values of descriptive statistics of expert evaluations, including mean values (Mean, Median) and their ranges (Minimum, Maximum), interquartile ranges (Lower Quartile, Upper Quartile), standard deviation (Std.Dev.), are given in Table 2, Table 3, Table 4 for all wines and separately for white and red wines.

**Table 2:** Descriptive statistics of expert evaluations for all wines.

	Descriptive Statistics (Expert)						
Variable	Mean	Median	Minimum	Maximum	Lower Quartile	Upper Quartile	Std.Dev.
Expert 1	84,917	85,000	67,000	95,000	82,000	88,000	5299
Expert 2	84,883	85,000	78,000	93,000	82,000	88,500	4126
Expert 3	81,167	81,000	68,000	89,000	80,000	82,000	3076
Expert 4	81,600	82,000	70,000	89,000	79,000	84,000	3679
Expert 5	83,150	83,000	77,000	94,000	82,000	85,000	2596
Expert 6	81,250	82,000	58,000	94,000	78,000	84,000	6920
Expert 7	83,033	83,000	77,000	90,000	81,000	85,000	3556
Expert 8	81,133	81,000	30,000	95,000	79,000	86,000	9118
Expert 9	81,750	82,000	60,000	88,000	79,000	85,000	4173
Expert 10	80,950	80,500	67,000	90,000	78,000	84,000	4470
Expert 11	82,067	82,000	68,000	90,000	80,000	84,500	4050

**Table 3:** Descriptive statistics of expert evaluations for white wines.

	Descriptive Statistics (Expert)Include cases: 1:20						
Variable	Mean	Median	Minimum	Maximum	Lower Quartile	Upper Quartile	Std.Dev.
Expert 1	87,200	88,000	77,000	95,000	83,500	90,000	4720
Expert 2	84,850	85,500	79,000	92,000	82,500	87,000	3731
Expert 3	80,900	80,000	78,000	87,000	79,000	82,000	2198
Expert 4	81,350	80,000	78,000	86,000	79,000	84,000	2870
Expert 5	82,200	82,000	77,000	86,000	81,000	84,000	2441
Expert 6	75,600	77,000	58,000	84,000	72,000	82,000	7437
Expert 7	82,700	83,000	77,000	89,000	79,000	85,000	3975
Expert 8	80,750	80,500	72,000	90,000	77,500	85,500	5300
Expert 9	84,200	84,000	82,000	87,000	83,000	85,000	1436
Expert 10	81,200	81,500	74,000	86,000	79,000	84,000	3350
Expert 11	82,700	83,000	78,000	87,000	81,000	85,000	2716

**Table 4:** Descriptive statistics of expert evaluations for red wines.

	Descriptive Statistics (Expert)Include cases: 21:60						
Variable	Mean	Median	Minimum	Maximum	Lower Quartile	Upper Quartile	Std.Dev.
Expert 1	83,775	85,000	67,000	94,000	81,500	87,000	5255
Expert 2	84,900	85,000	78,000	93,000	81,500	89,000	4355
Expert 3	81,300	81,000	68,000	89,000	80,000	83,000	3451
Expert 4	81,725	82,000	70,000	89,000	79,500	84,000	4051
Expert 5	83,625	84,000	78,000	94,000	82,500	85,000	2569
Expert 6	84,075	84,000	74,000	94,000	81,500	86,500	4576
Expert 7	83,200	83,000	77,000	90,000	81,000	85,000	3368
Expert 8	81,325	81,500	30,000	95,000	80,000	87,000	10,582
Expert 9	80,525	80,500	60,000	88,000	78,000	83,000	4552
Expert 10	80,825	80,000	67,000	90,000	78,000	84,000	4971
Expert 11	81,750	82,000	68,000	90,000	80,000	84,000	4573

The positional analysis of the results of organoleptic evaluation of the tested wine samples, carried out by the Reliability/Item Analysis module, allowed to calculate the Cronbach's alpha value equal to 0.843. This indicator, calculated according to the initial point scale taking into account its variability, made it possible to assess the contribution of each expert to the consistency of expert assessments. The closeness of Cronbach's alpha to 1 characterizes the reliability of the total score scale (column Sum, Table 1), hence the consistency of expert assessments, as high. Cronbach's alpha values, calculated with successive deletion of the assessments of experts 1, 2, 3,…, 11, allowed to determine the influence of each expert on the overall consistency of expert assessments. If Cronbach's alpha exceeds 0.843, then the expert reduces the overall consistency of expert assessments, otherwise increases it. Experts 1, 2, 3, 4, 5, 8, 9, 10, 11 were established to increase the overall consistency of assessments, while experts 6 and 7 reduced it.

During Reliability/Item Analysis module implementation, a matrix file of the Pearson pairwise correlation coefficients was formed characterizing relationships between expert evaluations (Table 5).

**Fig. 1:**
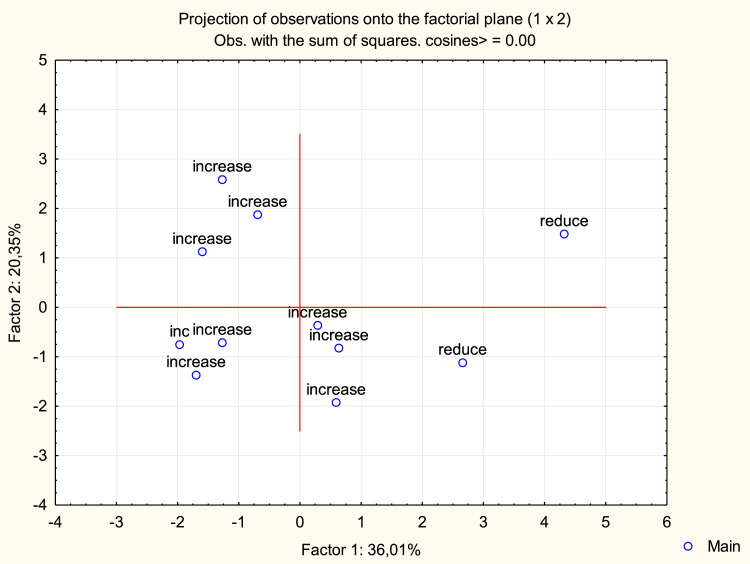
Scatterplot for experts.

Using pairwise correlation coefficients (Table 5) and designating the group of experts decreasing the consistency of evaluations as “reduce” (6, 7), while the group of experts increasing the consistency of evaluations as “increase”, it can be seen that experts form the groups of homogeneity (clusters) in relation to their contribution to the consistency of evaluations. As can be seen from Fig. 1 constructed by principal component analysis (PCA), experts increasing the consistency are located on the central and left parts of the diagram, while those, which decrease the consistency, are on the right part of it.

**Table 5:** Matrix file of pairwise correlations between experts.

	Expert 1	Expert 2	Expert 3	Expert 4	Expert 5	Expert 6	Expert 7	Expert 8	Expert 9	Expert 10	Expert 11
Expert 1	1000	0,381	0,437	0,532	0,414	0,072	0,174	0,465	0,433	0,249	0,426
Expert 2	0,381	1000	0,512	0,396	0,537	0,331	0,294	0,458	0,184	0,429	0,467
Expert 3	0,437	0,512	1000	0,620	0,559	0,397	0,345	0,674	0,545	0,606	0,546
Expert 4	0,532	0,396	0,620	1000	0,604	0,360	0,176	0,608	0,347	0,328	0,524
Expert 5	0,414	0,537	0,559	0,604	1000	0,550	0,337	0,487	0,299	0,379	0,307
Expert 6	0,072	0,331	0,397	0,360	0,550	1000	0,195	0,390	−0,013	0,343	0,148
Expert 7	0,174	0,294	0,345	0,176	0,337	0,195	1000	0,147	−0,068	0,308	−0,014
Expert 8	0,465	0,458	0,674	0,608	0,487	0,390	0,147	1000	0,533	0,579	0,569
Expert 9	0,433	0,184	0,545	0,347	0,299	−0,013	−0,068	0,533	1000	0,344	0,452
Expert 10	0,249	0,429	0,606	0,328	0,379	0,343	0,308	0,579	0,344	1000	0,357
Expert 11	0,426	0,467	0,546	0,524	0,307	0,148	−0,014	0,569	0,452	0,357	1000
Means	84,92	84,83	81,167	81,600	83,15	81,250	83,033	81,133	81,75	80,95	82,067
Std.Dev.	5299	4126	3076	3679	2596	6920	3556	9118	4173	4470	4050
No.Cases	60,00										
Matrix	1000

The reliability of the total scale of scores (column Sum) and average scores given by experts (column The average) were assessed (Table 6) by positional analysis of transposed Table 1. The aggregate of average scores given by experts (column The average) is defined as the loyalty scale of experts. With the increase in the average value, the loyalty increases, otherwise the loyalty decreases.

**Table 6:** The values of the sum of scores and average scores given by experts.

Expert number	Sum	The average
1	5095	84,917
2	5093	84,883
3	4870	81,167
4	4896	81,600
5	4989	83,150
6	4875	81,250
7	4982	83,033
8	4868	81,133
9	4905	81,750
10	4857	80,950
11	4924	82,067

Positional analysis of transposed data from Table 1 made it possible to evaluate the contribution of each of the 60 wine samples to the reliability of the loyalty scale (Table 7). Cronbach's alpha values after successive removal of wine samples from positional analysis allowed to isolate samples reducing/increasing the reliability of the loyalty scale. Samples 1, 12, 22, 25, 29, 32, 33, 34, 38, 42, 46, 47, 48, 49, 52, 53, 54, 55, 56, 60 (in bold italics) decrease the reliability of the loyalty scale, the rest – increase.

**Table 7:** Results of positional analysis for wine samples.

	Summary for scale: Mean=4941,27; Std.Dv.=87,2904; Valid N:11 (Expert tran)				
	Cronbach alpha: 0,869,019; Standardized alpha: 0,877,981;				
	Average inter-item corr.: 0,124,210				
variable	Mean if deleted	Var. if deleted	StDv. if deleted	Itm-Totl Correl.	Alpha if deleted
1	4860,273	7203,653	84,874	−0,652	0,876
2	4862,545	6526,248	80,785	0,431	0,865
3	4859,455	6662,976	81,627	0,407	0,866
4	4859,182	6491,421	80,569	0,645	0,862
5	4856,727	6605,834	81,276	0,732	0,863
6	4858,727	6659,835	81,608	0,410	0,866
7	4857,000	6806,546	82,502	0,278	0,868
8	4857,636	6640,776	81,491	0,568	0,864
9	4855,273	6376,380	79,852	0,762	0,860
10	4860,091	6378,264	79,864	0,519	0,863
11	4864,545	6517,338	80,730	0,320	0,868
12	4857,455	6896,430	83,045	0,102	0,869
13	4859,091	6536,083	80,846	0,448	0,864
14	4858,909	6586,992	81,160	0,406	0,865
15	4857,182	6313,239	79,456	0,863	0,858
16	4858,545	6666,066	81,646	0,522	0,865
17	4862,273	6695,289	81,825	0,437	0,866
18	4860,182	6485,239	80,531	0,680	0,862
19	4861,909	6684,810	81,761	0,300	0,867
20	4855,455	6356,793	79,730	0,865	0,859
21	4859,636	6653,686	81,570	0,725	0,864
22	4873,909	6184,446	78,641	0,274	0,880
23	4861,091	6470,810	80,441	0,622	0,862
24	4858,727	6566,562	81,034	0,702	0,863
25	4859,636	7017,867	83,773	−0,257	0,872
26	4857,636	6608,049	81,290	0,572	0,864
27	4860,091	6552,992	80,951	0,489	0,864
28	4859,182	6667,058	81,652	0,482	0,865
29	4859,545	6831,703	82,654	0,169	0,869
30	4858,000	6704,728	81,882	0,587	0,865
31	4859,545	6660,793	81,614	0,477	0,865
32	4857,364	6851,322	82,773	0,089	0,870
33	4858,818	6848,330	82,755	0,098	0,870
34	4858,545	6918,430	83,177	−0,004	0,871
35	4857,273	6442,562	80,266	0,811	0,860
36	4856,364	6745,868	82,133	0,309	0,867
37	4857,727	6592,380	81,193	0,636	0,863
38	4860,455	6832,612	82,660	0,171	0,869
39	4858,000	6582,000	81,130	0,530	0,864
40	4856,000	6732,363	82,051	0,287	0,867
41	4857,364	6764,414	82,246	0,283	0,867
42	4864,273	6608,744	81,294	0,261	0,869
43	4859,909	6499,901	80,622	0,665	0,862
44	4859,091	6708,627	81,906	0,380	0,866
45	4855,455	6778,430	82,331	0,182	0,869
46	4855,000	6938,728	83,299	−0,041	0,871
47	4857,182	7255,966	85,182	−0,530	0,878
48	4856,727	6965,289	83,458	−0,095	0,871
49	4853,091	6980,810	83,551	−0,100	0,873
50	4858,091	6592,265	81,193	0,633	0,863
51	4857,727	6803,653	82,484	0,358	0,867
52	4858,727	6944,925	83,336	−0,051	0,872
53	4859,000	7148,364	84,548	−0,436	0,875
54	4857,000	7162,545	84,632	−0,652	0,875
55	4852,636	6953,140	83,385	−0,068	0,871
56	4861,727	7044,199	83,930	−0,404	0,872
57	4859,000	6722,000	81,988	0,387	0,866
58	4858,727	6676,743	81,711	0,571	0,865
59	4858,909	6813,537	82,544	0,197	0,868
60	4865,455	6852,248	82,778	0,053	0,872

**Fig. 2:**
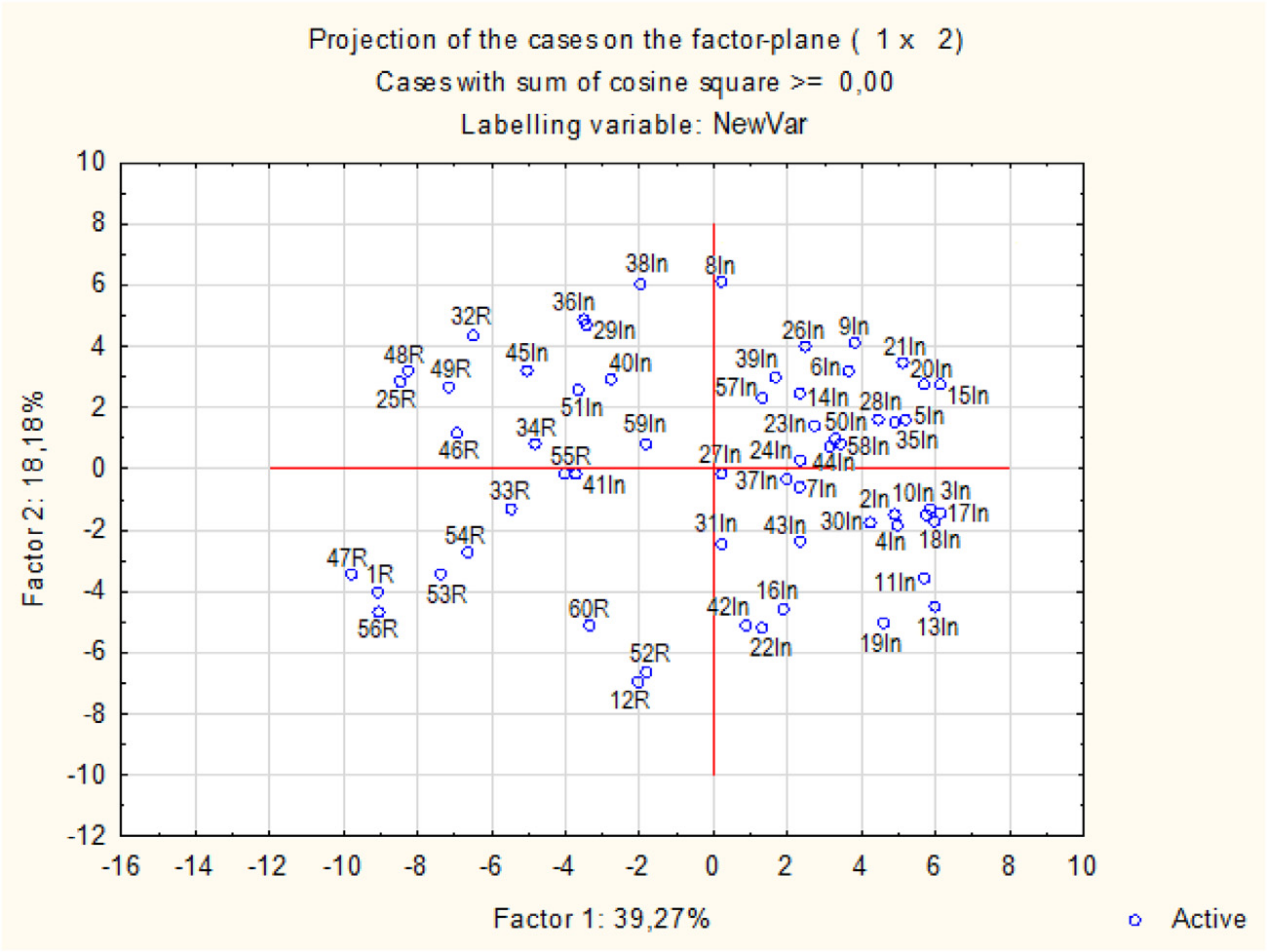
Scatterplot for wine samples.

Wine samples increasing and decreasing the reliability of the loyalty scale also have a cluster structure. Unfortunately, a large number of samples did not allow to apply PCA method for cluster structure illustration, therefore, discriminant analysis scatterplot is given in Fig. 2. Wine samples decreasing the reliability are predominantly localized on the left part of the chart, while wine samples increasing the reliability are predominantly localized on the right part of the chart.

## 2 Experimental Design, Materials and Methods

### 2.1 Research objects

60 samples of natural dry red and white grape wines produced in 2010–2015 in the territory of main wineries of Krasnodar region (Russia) were analyzed: “Myskhako”, “Fanagoria Number Reserve”, “Kuban-Vino”, “Southern wine company (SWK)”, “Villa Victoria”, “Chateau Tamagne”, “Chateau le Grand Vostock”. All the wine samples were produced according to traditional technologies from European (Cabernet, Merlot, Aligote, Riesling, Saperavi, etc.) and hybrid grape varieties (Bianca, Viorica, Moldova, Pervenets Magaracha, etc.) and were kindly provided for research by their manufacturers. The wines were poured into dark green glass bottles with screw caps and stored until use at 10 °C. All wine samples were dry, alcohol content varied from 9 to 13% (v/v) and pH values ranged from 3.61 to 3.79. Dissolved oxygen in wines was measured by the immersion of the probe before bottling in barrels, which was less than 1 mg/dm^3^.

Wines from European grape varieties obtained by traditional technologies without the use of sulfur dioxide were not considered, since this category significantly differs in taste from wines for which sulfiting was used.

### 2.2 Sensory analysis

All experimental studies related to sensory analysis were carried out by 11 specialists from the Federal Research Center for Horticulture, Viticulture, Winemaking (FSC HVW, Krasnodar, Russia). Participants are considered experts in the field of wine, work in the wine industry and have professional experience in sensory analysis.

The wine sample (50 cm^3^) was poured into each glass and covered with a Petri dish with diameter of 5.7 cm 30 min before the sensory evaluation. The tests were carried out in a well-lit tasting room with controlled temperature conditions. All samples were fed at 16–22 °С at tables with white napkins. Experts were prohibited to communicate during the sensory evaluation procedure. The wines were served in transparent tulip-shaped glasses with a volume of 220 dm^3^. After evaluating each sample, participants were asked to wait at least 30 s, cleanse their palettes with water and crackers. The intervals between tasting of each sample were 2 min. During each interval, experts rinsed their mouths with water. Experts evaluated each sample in triplicate during the working week.

The sensory evaluation results of wine quality were expressed on a scale from 50 to 100 according to the well-known rating system [12]. According to this system, any wine sample is given 50 points, and based on the results of the sensory evaluation, the following maximum points can be added: appearance – up to 5 points, aroma – up to 15 points, taste – up to 20 points, overall impression and capability of aging – up to 10 points. For a consolidated assessment of the organoleptic characteristics of wines, the average scores of sensory evaluations were used according to the results of tasting by a group of experts.

In Russia, official methods for the sensory evaluation of wines express the results as points or use descriptive characteristics in terms of organoleptic indicators (transparency, color, aroma, taste). Ten or 100-point score scales are used. The 100-point system is used, as a rule, at international tasting competitions.

### 2.3 Data analysis

All calculations were implemented using the STATISTICA software (v. 10) [16]. The pairwise consistency of experts was determined using Spearman's rank correlation coefficient, the “individual” consistency was established by the multiple correlation coefficient, group consistency – by means of Kendall's concordance coefficient and Cronbach's alpha criterion (Reliability and Item Analysis). However, the listed statistical criteria for the consistency of expert evaluations – Spearman's rank correlation coefficients, Kendall's and Kronbach's alpha correlations do not have generally accepted ranges of variation for their interpretation in the nominal scale, therefore, we focused on the degree of their proximity to 0 and 1. If the value of the criteria is closer to 0, the consistency is lower; following this trend, if the value is closer to 1, the consistency is higher. Scatter plots for experts and wine samples were built using the Multidimensional Scaling module.

## Ethical Statement

All procedures performed in studies involving human participants were in accordance with the ethical standards of the institutional and/or national research committee and with the 1964 Helsinki declaration and its later amendments or comparable ethical standards. All participants gave their consent in this experiment. No additional regulations were required.

## CRediT Author Statements

**Alexan A. Khalafyan:** Software, Formal analysis, Writing - Original Draft; **Zaual A. Temerdashev:** Conceptualization, Methodology, Writing - Review & Editing, Supervision, Project administration; **Vera. A. Akin'shina:** Software, Data analysis; **Yuri F. Yakuba:** Sensory analysis.

## Declaration of Competing Interest

The authors declare that they have no known competing financial interests of personal relationship that could have appeared to influence the work reported in this paper.
